# Evaluation of the Influence of Hypolipidemic Medication on Albino Wistar Rats’ Bone Tissue by NMR Diffusiometry

**DOI:** 10.3390/medicina60060918

**Published:** 2024-05-31

**Authors:** Emese Orban, Zsuzsanna Pap, Radu Fechete, Remus Sebastian Sipos

**Affiliations:** 1 Doctoral School of Medicine and Pharmacy, George Emil Palade University of Medicine, Pharmacy, Science, and Technology of Targu Mures, 38 Gheorghe Marinescu Str., 540142 Targu Mures, Romania; orban.emese@yahoo.com; 2 Department of Anatomy and Embryology, George Emil Palade University of Medicine, Pharmacy, Science, and Technology of Targu Mures, 38 Gheorghe Marinescu Str., 540142 Targu Mures, Romania; remus.sipos@umfst.ro; 3 Physics Department, Technical University of Cluj-Napoca, 28 Memorandumului Str., 400114 Cluj-Napoca, Romania; radu.fechete@phys.utcluj.ro

**Keywords:** osteoporosis, menopause, NMR diffusiometry, hypolipidemic medication

## Abstract

*Introduction:* The ongoing concern of the medical profession regarding chronic medication is related to increasing patient adherence and compliance to treatment and reducing medication side effects. In this respect, drugs represented by fixed-dose combinations of active substances within the same tablet have emerged. Such a principle can be extrapolated by following the potential beneficial effects that a chronic medication can have on chronic pathologies affecting different systems. *Materials and Methods:* The study included 48 female Albino Wistar rats, aged 16–18 months, which were divided into two groups: ovariectomized and non-ovariectomized rats. One batch of 12 non-ovariectomized rats received no treatment, becoming a control batch (NOVX-M). The ovariectomized (OVX) group was divided into 3 batches of 12 rats each: no treatment, control (OVX-M), fenofibrate-treated (OVX-F) and statin-treated (OVX-S) rats. At 12 weeks after ovariectomy, a femoral fracture occurred in the right hind limb of all animals included in the experiment To reveal the changes, at intervals of 2, 4, 6 and 8 weeks post-fracture, the proximal part of the femur was evaluated by NMR diffusiometry, which allows random motion of proton molecules expressed by self-diffusion coefficients, *D*, thus allowing analysis of the size and complexity of microscopic order cavities within biological structures, such as pores inside bones. *Results:* The effects of hypolipidemic medication in the absence of estrogen were evidenced, proving the beneficial effect that fenofibrate can have in preserving healthy tissue exposed to osteoporotic risk during the menopausal period. The effects of lipid-lowering medication are also influenced by the duration of administration. *Conclusions:* Osteoporosis and heart disease are two chronic pathologies that affect mainly female population in the second half of life, and proving the dual therapeutic potential of lipid-lowering medication may also have positive effects by increasing adherence and compliance to treatment.

## 1. Introduction

Bone tissue is defined as an active connective tissue that serves the human body through its function as structural support for muscle elements. It thus provides the functions of locomotion and support, while also protecting vital organs and nervous elements, storing minerals and growth factors as well as regulators of homeostasis; it is also the main site of hematopoiesis [1,2]. Humans’ (and all mammals’) bones are organized according to the resistance required to overcome the forces of physiological mechanical movements exerted on them [3]. The normal metabolic state of bone could be defined as the balance between osteoblastic and osteoclastic activity. An eloquent example of hormonal impairment of this homeostasis is the postmenopausal state of the female population. This postmenopausal state, through hormonal imbalance, causes an increase in osteoclastic activity at the expense of osteoblastic activity, intensifying the process of bone resorption [4]. Physiologically, bone remodeling occurs throughout life in an asynchronous manner in different locations of the entire human skeleton. This process is initiated by osteocytes, mature bone cells, incapable of cell division, which cause bone resorption by osteoclasts followed by production of the same amount of matrix by osteoblasts [5].

Bone fracture is a pathological condition of a bone element that involves the interruption of its continuity. It is one of the most common traumatic injuries in humans [6]. Osteoporosis is a relatively common skeletal disorder that predominantly affects postmenopausal women [7]. It is a silent disease, characterized by decreased bone density, and is very often diagnosed only when osteoporotic fractures occur [8]. The hormonal changes specific to the postmenopausal period leads to significant changes in the bone’s mineral density, thus affecting the quality of bone tissue, i.e., its mechanical resistance.

Primary osteoporosis, the most common cause of decreased bone mineral density, is classified into type I, postmenopausal osteoporosis, and type II, senile osteoporosis. Secondary osteoporosis is caused by hypercortisolism, hyperthyroidism, hyperparathyroidism and immobility [9]. Primary osteoporosis occurs as a result of three major events: failure to reach optimal peak mass around age 20, old age through decreased serum levels of vitamin D, calcium and protein, and excessive bone degradation caused physiologically by the decline in menopausal oestradiol [10].

Another major health problem at postmenopausal age is dyslipidemia, a pathology manifested by altered lipid metabolism, which is one of the main causes of atherosclerotic disease. Osteoporosis and atherosclerotic disease, in addition to being promoted by advancing age, are degenerative processes with common pathogenic mechanisms related to bone and vascular mineralization [11]. Dyslipidemia is a pathological condition involving alterations in the lipid profile through increased total cholesterol, LDL cholesterol (low-density lipoprotein) or triglycerides, or a reduced plasma concentration of HDL cholesterol (high-density lipoprotein) or a combination of the above [12].

Statin-indicated pathologies include all atherosclerotic cardiovascular disease, but also other high-risk conditions requiring primary prevention despite the absence of documented atherosclerotic cardiovascular disease [13]. Statins have remained one of the most effective methods of reducing LDL cholesterol by inhibiting 3-hydroxy-3-methylglutaryl-coenzyme A (HMG-CoA) reductase, the enzyme responsible for endogenous cholesterol synthesis [14]. The efficacy of statins is documented, with monotherapy with an HMG-CoA reductase inhibitor being able to lower LDL cholesterol levels by approximately 30–50% [15]. In addition to the beneficial hypolipidemic effect, statins have vasodilatory, antioxidant, anti-inflammatory and even immunosuppressive effects [16]. However, a study in Vienna concludes that there is an increased incidence of osteoporosis among people treated with high doses of statins as opposed to the lower-dose group [17], which highlights the importance of monitoring statin medication in patients at risk of osteoporosis.

Another subclass of drugs used to control the lipid profile is the fibrates. The mechanism of action of fibrates is to stimulate the expression of PPAR-alpha (peroxisome proliferator activated α-receptors) receptors, a key transcription factor that induces the expression of several proteins involved in lipoprotein metabolism, such as lipoprotein lipase, apolipoprotein A1 and apolipoprotein A2, respectively [18]. The results of treatment with fibrates are notable for their ability to decrease triglyceride levels by up to 50% and LDL cholesterol by up to 20% and increase HDL cholesterol levels by up to 20% [19].

Increased cholesterol levels in the blood are reflected in the bone by an increased amount of cholesterol in the bone marrow and can lead to decreased bone mineral density, increased bone resorption, significant loss of cortical and trabecular bone mass, decreased levels of osteocalcin, a marker of osteoblastic activity, and increased levels of deoxypyridinoline, a marker of osteoclastic activity. Thus, untreated dyslipidemia can certainly become a risk factor for the development of osteoporosis [20]. At the same time, dyslipidemia has been associated with the postmenopausal period, with a correlation found between low bone mineral density and increased adipocytes in the bone marrow [21].

Dyslipidemia is a risk factor for cardiovascular disease, the leading cause of death worldwide. This risk factor is addressed from the perspective of the therapeutic targets set out in the cardiovascular disease prevention guidelines. These therapeutic targets, once updated, have proposed increasingly lower serum lipid levels, which is why the use of lipid-lowering medication has increased not only in terms of the number of people using it, but also in terms of the duration of administration, which has increased, as has the dosage. This medication, as studies show, intervenes on the two cell lines involved in the processes of osteogenesis and osteolysis.

These lipid-lowering drugs also have important effects on bone metabolism. Lipophilic statins, such as simvastatin, increase the activity of alkaline phosphatase, which is a marker of early osteoblastic differentiation, and at the same time increase the expression of osteocalcin, a marker of late osteoblastic differentiation of bone protein and type I collagen. On the other hand, according to the literature, fibrates stimulate preosteoblast proliferation and osteoprotegerin release, the latter being an important inhibitor of osteoclast differentiation. These findings indicate that fibrates may have an anti-resorptive effect. On the other hand, fibrates stimulate osteoblast differentiation and proliferation, thereby promoting bone matrix formation [22,23].

As a working method, we used ^1^H NMR diffusiometry, a method intensively developed for the study of porous materials such as rocks or biomaterials, in particular bones, being based on the use of water molecules inside the pores as spy molecules interacting with the pore’s walls, thus providing information about their geometry, in particular about the size of the pores and their distribution. NMR diffusiometry allows obtaining the undergoing a random motion of (water) proton expressed by the self-diffusion coefficients, *D*, (or the distribution of self-diffusion coefficients), thus allowing for the analysis of the size and intricacy of microscopic order cavities within biological structures [24].

In general, one can speak of two types of diffusion, namely in the presence of a concentration gradient of a type of molecule in a fluid (because it implies its mobility) and in the absence of this concentration gradient and which is called self-diffusion.

In medicine, the method of nuclear magnetic resonance (NMR) diffusiometry is used to detect abnormalities in tissues and assess the condition of various organs. In the study of human bone tissues, the measurement of the distribution of the MRI self-diffusion coefficient has been used to characterize bone porosity and bone mineral density (BMD). An example of such a study was performed by Techawiboonwong et al. (2008), who used this method to assess BMD changes in human bones with osteoporosis. Their results showed that the distribution of the self-diffusion coefficient can be used to differentiate healthy bones from those with osteoporosis [25].

The aim of the study is to highlight, by a modern method based on the measurement of the distribution of the self-diffusion coefficient of water molecules by nuclear magnetic resonance (^1^H NMR), how the hypolipidemic medication, intensively used in cardiovascular disease prophylaxis, can interfere with the biology of bone tissue and the impact it can have on osteoporosis processes. NMR diffusiometry was chosen to assess the porosity of bone tissue, because although it is a little-used method to determine the degree of bone damage, it allows for the measurement of specific parameters at the molecular level sensitive to the displacement of water molecules within bone microcavities.

## 2. Materials and Methods

The study is based on an experiment with 48 female Albino Wistar rats aged 16–18 months, equivalent to the perimenopausal period in females (approximately 47–52 years), a period characterized by an imbalance between osteoclastic and osteoblastic activity, with the osteoclastic activity being higher and thus the bone resorption process being more intense. The average mass of the rats was 300 g. The intervention that made it possible to obtain a biological model of induced osteoporosis was ovariectomy. A total of 36 rats underwent the ovariectomy procedure, thus moving into an iatrogenically induced menopausal period, and the remaining 12 laboratory animals did not undergo this intervention, remaining in the perimenopausal period. The non-ovariectomized rat group (NOVX) did not receive any treatment and became the control group of non-ovariectomized rats (NOVX-M). The group of ovariectomized rats (OVX) was divided into 3 batches of 12 rats each: ovariectomized rats without treatment, control (OVX-M) and ovariectomized rats given fibrate (OVX-F) and statin (OVX-S). At 12 weeks after induction of menopause in the ovariectomized group, a femoral fracture occurred in the right femur of all animals included in the experiment. Both drugs were administered by gavage at a dose of 10 mg/kg/day starting from the moment of fracture. The ovariectomy and fracture induction procedures were performed under anesthesia by intraperitoneal injection of ketamine and xylin at a dose of 4–5 mg/kg.

At 2, 4, 6 and 8 weeks after fracture, a variable number of rats from each of the 4 batches described above were euthanized by an overdose of ketamine and xylin (8–10 mg/kg) to harvest contralateral femurs, biological material subsequently used in the evaluation of bone tissue by the ^1^H NMR diffusiometry method. Each harvested femur was kept in formaldehyde for 30 days, sufficient time to allow migration of molecular water into all microcavities of osteoporotic and osteopenic bone tissue, respectively. For the detection of changes in bones only the proximal parts of the femoral bones including the femoral head, the femoral neck and the proximal part of the femoral diaphysis were considered. Before each measurement, the excess water caused by excess formaldehyde was blotted with absorbent paper in order not to interfere with the results of the experiment.

**Figure 1:**
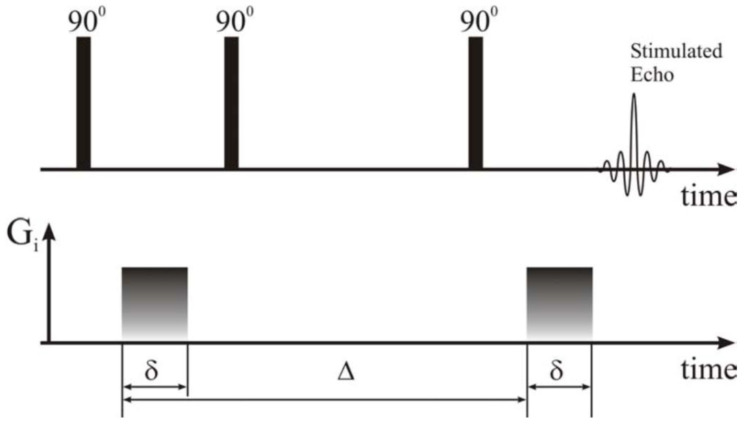
Pulsed gradient stimulated echo—the sequence of pulsed pulses used in determining the self-diffusion coefficient [26].

Physically, such a method is based on nuclear magnetic resonance (NMR) measurements of the distribution of the self-diffusion coefficients of molecules, mainly water filling the pores. One such method is that denoted by PGSE (pulsed gradient stimulated echo), for which we have a pulse sequence consisting of three 90-degree pulses producing a stimulated echo (see Figure 1) [26].

The amplitude of the stimulated echo is given by the variable amplitude of a pair of magnetic field gradients with duration δ and a diffusion time, Δ between them by encoding the position of the molecules. From a physical point of view, we obtain a phase encoding of the NMR signal given by the variable precessing motion of the nuclear spins of ^1^H from molecules into a magnetic field. The magnetic field gradient produces in space a variation of the magnetic field, in other words, a variation of the precessing velocity of the nuclear spins encoding then the position with the first gradient of magnetic field and decoding the (new) position by applying the second gradient. The diffusion time Δ allows that the liquid molecules (here water) to explore (by self-diffusion) the free space surrounding them and, in pores, to interact with them. In this way, one has so-called spy molecules which may experience a free self-diffusion in large pores and a restricted diffusion in small pores. Therefore, the so called apparent self-diffusion coefficient *D* can give us information related to pore’s size.

Further, after obtaining the experimental data, i.e., the normalized value of the NMR signal to the value of the measured signal in the absence of the magnetic field gradient, an inverse Laplace transform can be applied. The decay curve is obtained as a function of the parameter ***q***

(1) q=γGδ,

such as [27,28]:

(2) $$\frac{S\left(q\right)}{S\left(0\right)}=\int f\left(D\right)exp\left\{-q^{2}D\left(∆-\frac{\delta }{3}\right)\right\}dD,$$

described by ***γ*** the gyromagnetic ratio by ***G*** the amplitude of the gradient and by Δ its duration in time, in which the distribution function *f*(*D*), also called Laplace function, usually denoted or plotted on the vertical axis, can also be called the normalized probability of having molecules with a given value of the self-diffusion coefficient, *D*.

For the experimental measurement, the Bruker Minispec MQ 20 MHz NMR spectrometer with a pulsed gradient unit with a maximum value of *G*_x,max_ = 3.54 T/m was used. It was calibrated by measuring at 40 °C the self-diffusion coefficient of the water-diluted copper sulphate solution. The proximal portion of the rat femur was placed in a 10 mm diameter NMR glass tube (special glass test tube that does not contain ^1^H) specially made for use in NMR measurements. The tube was sealed with a rubber cap and 100% relative humidity was ensured so as not to interfere with the self-diffusion process of water molecules. To obtain the distributions of the self-diffusion coefficients *f*(*D*) (or Laplace spectra), a specially developed program was used [27,28,29,30], which is based on the inverse Laplace transform of the NMR signal.

The Scientific Research Ethics Committee of the University of Medicine, Pharmacy, Sciences and Technology “George Emil Palade” in Targu Mures approved the experiment and study protocols according to document no. 1186/19 November 2020. The medical and surgical interventions were performed following the approval of the experiment and study protocols by the Ethics and Deontology Commission of the University of Medicine and Pharmacy of Targu Mures, in accordance with documents no. 2/2009, 29/26 June 2012.

## 3. Results

**Figure 2:**
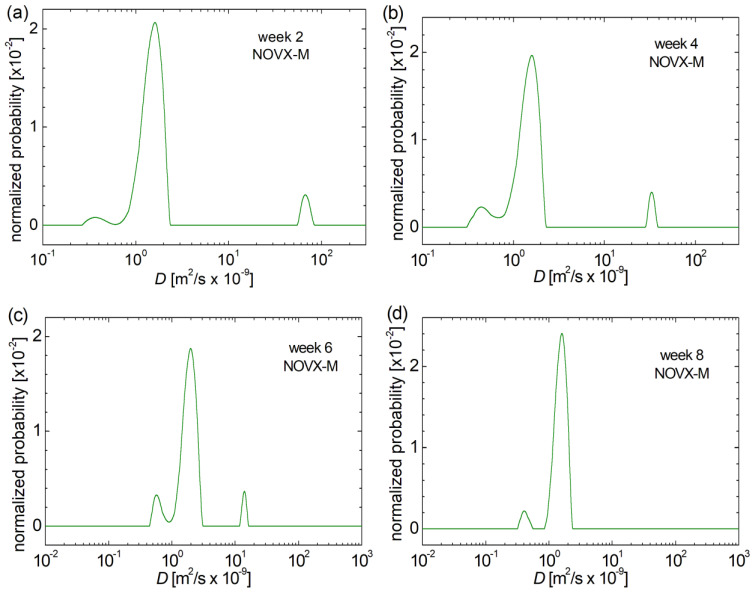
Evolution of bone pore size in the (**a**) control batch of non-ovariectomized rats at perimenopausal age for 2 weeks post-fracture, (**b**) control batch of non-ovariectomized rats at perimenopausal age for 4 weeks post-fracture, (**c**) control batch of non-ovariectomized rats at perimenopausal age for 6 weeks post-fracture, (**d**) control batch of non-ovariectomized rats at perimenopausal age for 8 weeks post-fracture, evaluated from the distribution of self-diffusion coefficient *D*.

The measured distributions of the self-diffusion coefficient *D* for the control batch of non-ovariectomized rats (NOVX-M) are presented in Figure 2 for 2, 4, 6 and 8 weeks after the fracture occurred. Three distinctive peaks can be seen, which can be associated with water molecules diffusing in pores with different diameters. As a simplest association, one can consider that this NMR experiment can describe the following: (i) small pores associated with the peaks located at the smallest value of *D* (usually below 10^−9^ m^2^/s); (ii) medium size pores associated with the peaks located at the *D* values usually between 1 and 2.5 × 10^−9^ m^2^/s; and (iii) large pores associated with the peaks located at the *D* values usually larger than 2.5 × 10^−9^ m^2^/s. The amount of water filling each type of pores (sometimes named as pools or ^1^H reservoirs) is proportional to the peaks integral area (area under the curve). Thus, one can observe a relatively small number of water (10% formalin) molecules filling the small and large pores, while the largest amount of water fills the medium-sized pores. The Laplace spectra resolution (degree of separation of peaks in the distribution of *D*) can be a clue of physically separation of the previously discussed types of pores from femoral bone. For example, the peaks associated with the small pores (the small peak located at smallest values of *D*) are clearly separated in week 8 (Figure 2d), while in the other weeks, the tendency of overlapping with peaks associated with medium-sized bone pores is visible. From a quantitative point of view, but also from the point of view of the value of the self-diffusion coefficient, an increase in the amount of small pores is observed in week 4 (Figure 2b), but in perimenopausal week 8, a slight regression in the amount of small pores is observed. Medium-sized pores remain relatively constant in size over the 8 weeks. A gradual shrinkage of large pores is evident from perimenopausal week 2 (Figure 2a) to week 6 (Figure 2c), up to their disappearance in perimenopausal week 8.

**Figure 3:**
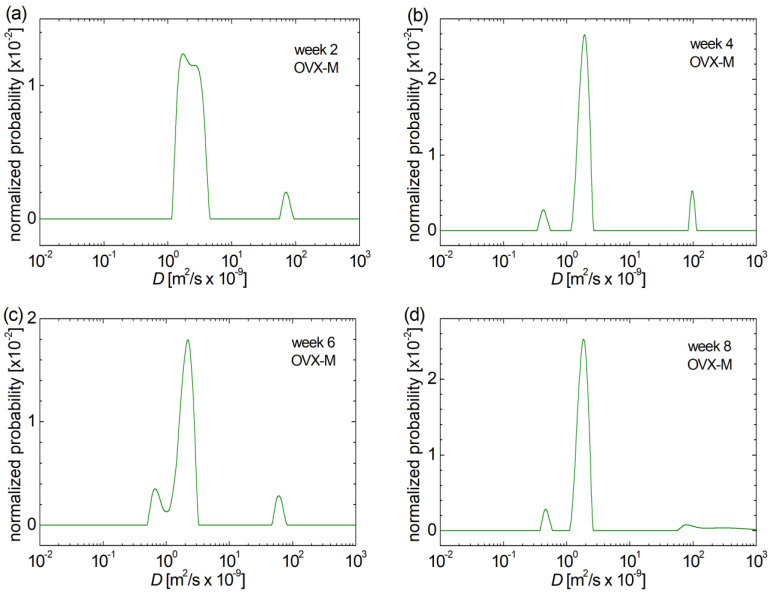
Evolution of bone pore size in the (**a**) control batch of ovariectomized rats during induced menopause for 2 weeks post-fracture, (**b**) control batch of ovariectomized rats during induced menopause for 4 weeks post-fracture, (**c**) control batch of ovariectomized rats during induced menopause for 6 weeks post-fracture, (**d**) control batch of ovariectomized rats during induced menopause for 8 weeks post-fracture, evaluated from the distribution of self-diffusion coefficient *D*.

The graph representing the diffusion coefficient *D*-distribution measured for the femoral samples belonging to rats in the second week post-fracture in the ovariectomized group—control batch (OVX-M) of menopause-induced rats (Figure 3a) illustrates the absence of small pores, the existence of well-defined large pores and the existence of an overlap of peaks associated with two types of medium pores. In the fourth week (Figure 3b), there are three categories of well-individualized pores: small, medium, and large, respectively. The small pores show a tendency to migrate to medium pores in the sixth week, and the large pores are smaller compared to those observed in the week 4 post-fracture.

Large pores increased in size in week 4 post-fracture (Figure 3b), decreased in size in week 6 but became more heterogeneous (Figure 3c), and in the eighth week (Figure 3d), they decreased quantitatively and became much more heterogeneous, as can be observed from the distributed peaks located at the largest values of *D*. In the sixth week, the position of the peak associated with the medium sizes pores shifted slightly towards larger values, showing that the size of the medium pores increases slightly (correlated with the decreased size of large pores), and then decreases slightly in the eighth week post-ovariectomy (correlated to the increase in heterogeneity of the large pores).

**Figure 4:**
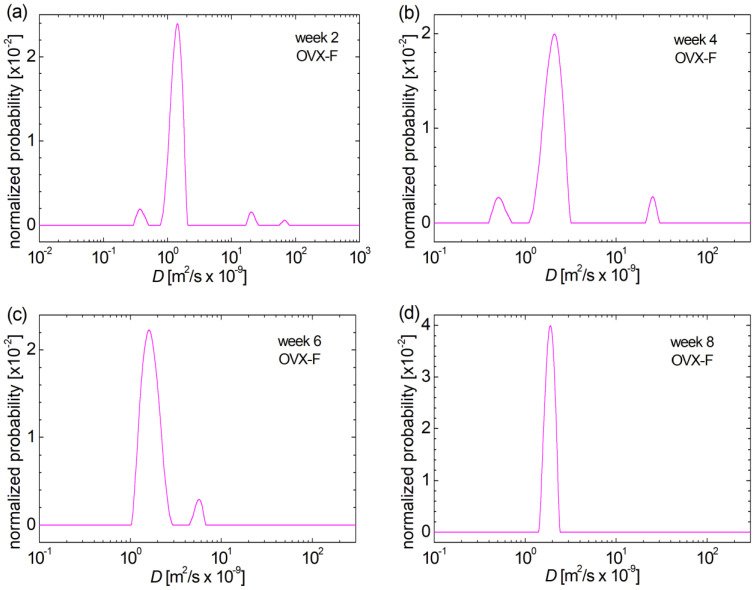
Evolution of bone pore size in the ovariectomized group of rats during induced menopause and treatment with fibrates (**a**) 2weeks post-fracture, (**b**) 4 weeks post-fracture, (**c**) 6 weeks post-fracture and (**d**) 8 weeks post-fracture evaluated from the distribution of self-diffusion coefficient *D*.

In the case of the large pores, characteristic to the femoral bone of ovariectomized rat batch treated with fibrates (OVX-F), one can observe from the appearance of a secondary peak associated to large pores that the effect of this treatment, at least in the early stage, has a negative impact on bone structure (see Figure 4a). Nevertheless, one can mention also a benefit of the treatment with fibrates, as can be observed for aged rats (starting from week 6 post-fracture), that there is a progression in the sense that the larger pores present an evolution towards medium-sized pores, with no large pores detected in week 8 (Figure 4d). A similar evolution that was found for lager pores can be observed also for the small pores. First, from the integral area, one can deduce that numerous small pores are formed in week 4 (Figure 4b), which, after two more weeks of treatment, disappear entirely (Figure 4c), leading to a complete disappearance of small and large pores in week 8 post-fracture due to the treatment with fibrates (Figure 4d). Thus, at week 8 post-fracture, the only remaining pore category is medium-size pores leading to a unique narrow peak in the *D*-distribution. This indicates that the pores are more homogeneous; having smaller pore’s diameters compared to the mean pore’s diameters measured in weeks 2, 4 and 6 for the upper epiphysis of the femoral bone for this batch (OVX-F).

**Figure 5:**
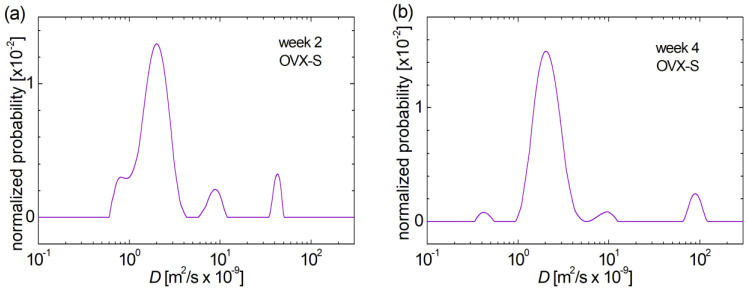

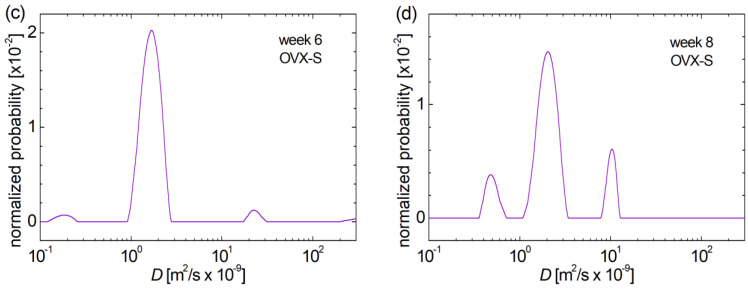
Evolution of bone pore size in the ovariectomized group of rats during induced menopause and statin treatment (**a**) 2 weeks post-fracture, (**b**) 4 weeks post-fracture, (**c**) 6 weeks post-fracture and (**d**) 8 weeks post-fracture, evaluated from the distribution of self-diffusion coefficient *D*.

Statin treatment resulted in wider distribution curves of the self-diffusion coefficient *D*. This denotes a larger heterogeneity of pore’s diameter for all categories of pores. Moreover, the small pores present a fluctuating evolution in time after ovariectomy and treatment. This peak presents a characteristic diffusion coefficient similar to those observed for the main peak, i.e., this presence can be observed only as a left shoulder overlapping the main peak. It seems that this overlap observed for distribution of self-diffusion coefficient in week 2 is mainly due to the menopause induced by ovariectomy (see Figure 3a and Figure 5a), while the treatment acts to reduce this effect, more efficient for fibrates (see Figure 4a). The shift towards smaller *D*-values indicates that the small pores decrease in size in weeks 4 (Figure 5b) and 6 (Figure 5c) after fracture and treatment, respectively, and then increase in diameter in week 8 (Figure 5d). The measured self-diffusion coefficient distribution curves show that the medium pores are slightly reduced in size only at 6 weeks, compared to those measured for the upper femoral epiphysis harvested at 2, 4 and 8 weeks post-fracture. One can observe that there are two resolved peaks of the diffusion coefficients assigned to the large pores of upper femoral epiphysis harvested at week 2 (Figure 5a); thus, it can be confirmed that there are two distinct size categories of large cavities an effect which was also observed in the case of treatment with fibrates of ovariectomized rats. Contrary to the treatment with fibrates, the treatment with statin leads to a slow evolution and a similar distribution of the self-diffusion coefficient with four peaks (two of them associated to large pores) was also measured for the upper femoral epiphysis harvested at week 4 post-fracture (Figure 5b). Nevertheless, the evolution is evident and can be quantified as follows: (i) a smaller peak (thus number) of larger pores presenting the smaller diameter but located in the same position as the peak measured for the samples from week 2 and (ii) a shift towards larger *D*-values for the peak associated with the largest pores than presenting a larger cavity diameters compared to those of rats from the same group (OVX-S) in week 2 after fracture. In week 6 post-fracture, the distribution of self-diffusion coefficient presents a well-known pattern with only 3 resolved peaks thus three distinct types of pores: small, medium and large (Figure 5c). The effect of statin was that in this case one observes the small pores with the smallest average diameter. A significant evolution is observed from week 6 to week 8 post-fracture of rats treated with statins. While the natural tendency (and the fibrate treatment) is that at week 8 perimenopausal (Figure 2d)/post-fracture (Figure 4d), the large pore to be absorbed in the case of the treatment with statin the larger pores are formed with the largest efficiency (Figure 5d), at the same time, the smallest pores are formed into a larger quantity. Moreover, these pores also present a large heterogeneity while they remain separate.

## 4. Discussion

The study demonstrated that lipid-lowering medication alters the organic matrix of bone tissue influencing the progression of the osteoporotic process, as demonstrated by pore development in the proximal end of the rat femur. Measurement of the self-diffusion coefficient of water molecules by ^1^H NMR allowed the observation of the effects of hypolipidemic treatment, which were correlated with the duration of treatment. The additional benefits of the method are due to the fact that this method provides data related to bone microarchitecture, i.e., the bones pores’ structure, while DEXA osteodensitometry only provides information about the bone mineral density.

From the effect of medication on bone tissue homeostasis point of view, this study showed that simvastatin caused a reduction in bone density at 8 weeks after the start of administration in ovariectomized rats superior to that caused by ovariectomy alone, whereas when talking about the process of callus formation in these groups after 8 weeks of treatment, fenofibrate showed a beneficial effect on the callus forming process and simvastatin delayed it [31]. The fact that in ovariectomized rats, a beneficial effect of hypolipidemic medication administration on bone density, especially fenofibrate, has been observed in the case of medium-term administration after 6 weeks, could explain the beneficial effect of fenofibrate on the callus formation process [31], since normally after week 6, one can discuss the remodeling of the primary callus.

Interpretation of the effects of hypolipidemic medication, simvastatin and fenofibrate, and relating these results to the data in the literature should be completed taking into account not only the presence or absence of estrogens, but also the type of bone tissue to which we refer, i.e., compact or cancellous bone tissue. Also, the duration of treatment administration may result in different effects on both bone tissue structure and callus process [22,32].

Studies in the literature are contradictory. Using visible spectroscopy, the effect of hypolipidemic medication on bone fracture healing in the absence and presence of estrogen was investigated. It was found that in non-ovariectomized rats, hypolipidemic medication with simvastatin and fenofibrate delayed the callus formation process, while with fenofibrate, this process was delayed much more. On the other hand, in the ovariectomized group of rats, the effects of the lipid-lowering drugs are opposite, so fenofibrate favors the callus formation process and simvastatin delays it [33].

Another study, which examined the effects of lipid-lowering drugs from histological images and T2 distributions, found that simvastatin and fenofibrate have negative effects on healthy trabecular bone. In osteoporotic bone, however, both medications have positive effects by increasing the percentage of femoral diaphysis trabecular bone [34]. These results are consistent with other studies too [35,36].

In the non-ovariectomized control group, a cyclical evolution of the bone genesis and resorption process can be observed. If the assessment at 2 weeks would be considered as the benchmark of the evaluation, it can be observed that it is followed by a more intense process of osteogenesis with an increase in bone density at 4 weeks. This process of osteogenesis is followed by an exacerbated process of osteolysis at 6 weeks, to return at 8 weeks to a bone density identical to the initial assessment at 2 weeks. This cycle could be considered an illustration of how bone tissue homeostasis is maintained, if we are talking about healthy bone. In the ovariectomized group, a steady increase in bone porosity can be observed, with the same exacerbation of osteolysis processes at 6 weeks followed by an increase in bone density at 8 weeks. These processes in the two control batches occur identically only at different rates, with the porosity of bone harvested from rats in the non-ovariectomized control group being lower all the time than that determined on bone from the ovariectomized control group.

Measurement of the self-diffusion coefficient of water molecules by proton NMR allows not only an assessment of the degree of osteoporosis, but also to obtain a distribution of the self-diffusion coefficient that more accurately reflects the architecture of bone tissue on a microscopic scale. The method is used to determine what kind of effects that various medications may have on both, the organic and the inorganic components of bone tissue. The use of innovative methods in the evaluation of biological tissues demonstrates the importance of multidisciplinary collaborations, but especially the benefits that the use of physical and chemical methods, combined with computer software, can bring to the advancement of medicine. Connecting different medical specialties with fundamental research methods can offer new perspectives in the management of the treatment of patients with multiple pathologies.

## 5. Conclusions

The effects of hypolipidemic drugs, in the absence of oestrogens, on the porosity of the proximal femoral end, depend on the time of administration and the type of medication. In the very short term, fibrates cause an increase in bone density and statins cause an increase in bone porosity. Medium-term administration results in a beneficial effect on bone density, especially in the case of fenofibrate. Short- and long-term administration causes a greater reduction in bone density than that caused by ovariectomy alone.

The results of the study might suggest that osteogenesis and osteolysis do not occur simultaneously and that osteogenesis would induce osteolysis which, when exacerbated, would induce new osteogenesis processes to restore balance, which is possible in the presence of oestrogens, i.e., in the absence of already established osteoporosis. The appearance of a large loss of bone mass is noted shortly after ovariectomy, and the loss increases in the long term, even if after the initial loss, a period of equilibrium occurs between the processes of osteogenesis and osteolysis.

## Data Availability

The original contributions presented in the study are included in the article, further inquiries can be directed to the corresponding author.

## List of Abbreviation

NOVX	Non-ovariectomized
OVX	Ovariectomized
M	Control
F	Fibrate
S	Statin
NMR	Nuclear magnetic resonance
LDL	Low-density lipoprotein
HDL	High-density lipoprotein
BMD	Bone mineral density
PGSE	Pulsed gradient stimulated echo
DEXA	Dual-energy X-ray absorptiometry
